# 1-(2,3,4,5,6-Penta­methyl­benz­yl)-2-(pyridin-2-yl)-1*H*-benzimidazole

**DOI:** 10.1107/S1600536814007934

**Published:** 2014-04-16

**Authors:** Fırat Anĝay, Ömer Çelik, Orhan Barlık, Mahmut Ulusoy

**Affiliations:** a Department of Physics, Institute of Sciences, Dicle University, 21280, Diyarbakır, Turkey; b Department of Physics, Faculty of Education, Dicle University, 21280, Diyarbakır, Turkey; c Science and Technology Application and Research Center, Dicle University, 21280, Diyarbakır, Turkey; d Department of Chemistry, Faculty of Science & Art, Harran University, 63300, Şanlıurfa, Turkey

## Abstract

In the title compound, C_24_H_25_N_3_, the benzimidazole ring system is essentially planar, with an r.m.s. deviation of 0.017 Å, and forms dihedral angles of 7.81 (5) and 87.61 (4)° with the pyridine and benzene rings, respectively. An intra­molecular C—H⋯N hydrogen bond is observed. In the crystal, mol­ecules are stacked along the *a* axis by weak C—H⋯π inter­actions.

## Related literature   

For the use of 2-(2-pyrid­yl)benzimidazole in coordination chemistry, see: Sahin *et al.* (2010 ▶); Harkins *et al.* (1956 ▶); Chiswell *et al.* (1964 ▶); De Castro *et al.* (1991 ▶); Maekawa *et al.* (1994 ▶); Khalil *et al.* (2001 ▶); Boca *et al.* (1997 ▶). For the use of N—N-type ligand systems involving 2,2′-bi­pyridine, see: Lippert (1999 ▶); Wong & Giandomenico (1999 ▶), Kelland & Farrell (2000 ▶). For related structures, see: Çelik *et al.* (2007 ▶, 2009 ▶, 2014 ▶).
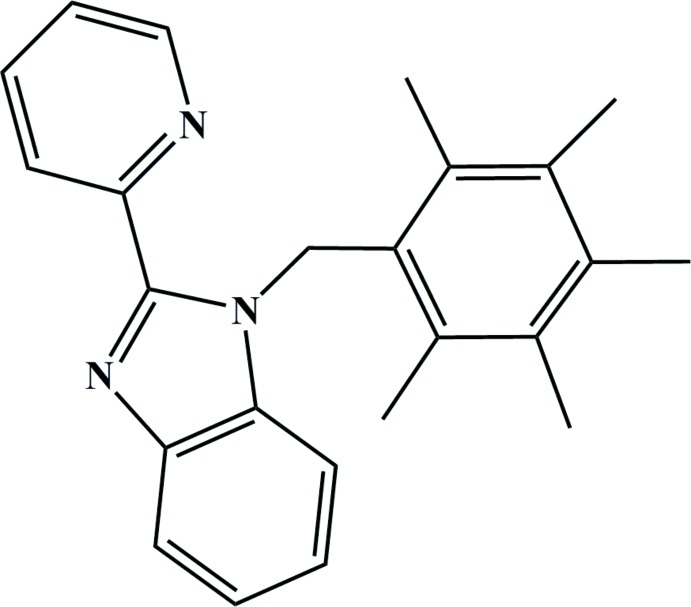


## Experimental   

### 

#### Crystal data  

C_24_H_25_N_3_
*M* *_r_* = 355.47
Monoclinic, 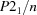

*a* = 5.3470 (3) Å
*b* = 21.0622 (12) Å
*c* = 17.0379 (9) Å
β = 97.699 (3)°
*V* = 1901.50 (18) Å^3^
*Z* = 4
Mo *K*α radiation
μ = 0.07 mm^−1^
*T* = 296 K
0.25 × 0.20 × 0.15 mm

#### Data collection  

Bruker APEXII CCD diffractometer
Absorption correction: multi-scan (Blessing, 1995 ▶) *T*_min_ = 0.982, *T*_max_ = 0.989
25354 measured reflections
3741 independent reflections
3056 reflections with *I* > 2σ(*I*)
*R*_int_ = 0.026

#### Refinement  

*R*[*F* ^2^ > 2σ(*F* ^2^)] = 0.052
*wR*(*F* ^2^) = 0.150
*S* = 1.04
3741 reflections
254 parameters
H-atom parameters constrained
Δρ_max_ = 0.24 e Å^−3^
Δρ_min_ = −0.17 e Å^−3^

### 

Data collection: *APEX2* (Bruker, 2007 ▶); cell refinement: *SAINT* (Bruker, 2007 ▶); data reduction: *SAINT*; program(s) used to solve structure: *SHELXS97* (Sheldrick, 2008 ▶); program(s) used to refine structure: *SHELXL2013* (Sheldrick, 2008 ▶); molecular graphics: *ORTEP-3 for Windows* (Farrugia, 2012 ▶); software used to prepare material for publication: *WinGX* (Farrugia, 2012 ▶).

## Supplementary Material

Crystal structure: contains datablock(s) I, global. DOI: 10.1107/S1600536814007934/rz5115sup1.cif

Structure factors: contains datablock(s) I. DOI: 10.1107/S1600536814007934/rz5115Isup2.hkl

Supporting information file. DOI: 10.1107/S1600536814007934/rz5115Isup3.cml

CCDC reference: 996309

Additional supporting information:  crystallographic information; 3D view; checkCIF report

## Figures and Tables

**Table 1:** Hydrogen-bond geometry (Å, °) *Cg* is the centroid of the C7–C12 benzene ring.

*D*—H⋯*A*	*D*—H	H⋯*A*	*D*⋯*A*	*D*—H⋯*A*
C13—H13*A*⋯*Cg* ^i^	0.97	2.91	3.6941 (18)	139
C13—H13*B*⋯N1	0.97	2.30	3.029 (2)	131

Symmetry code: (i) 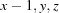.

**Fig. 1.**
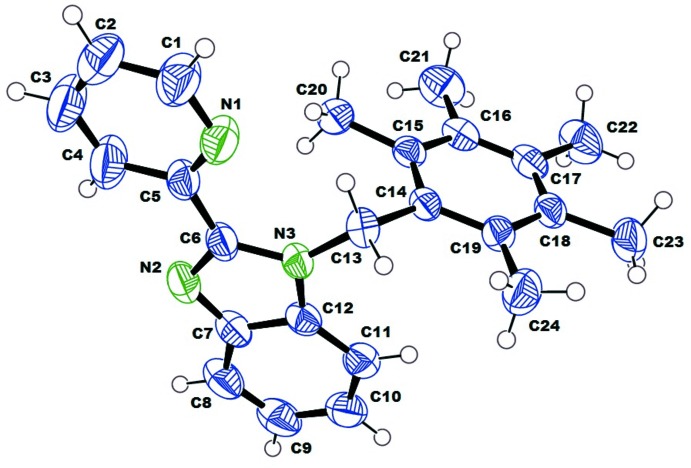
ORTEP-3 of the title compound, showing displacement ellipsoids drawn at 50% probability level.

**Fig. 2.**
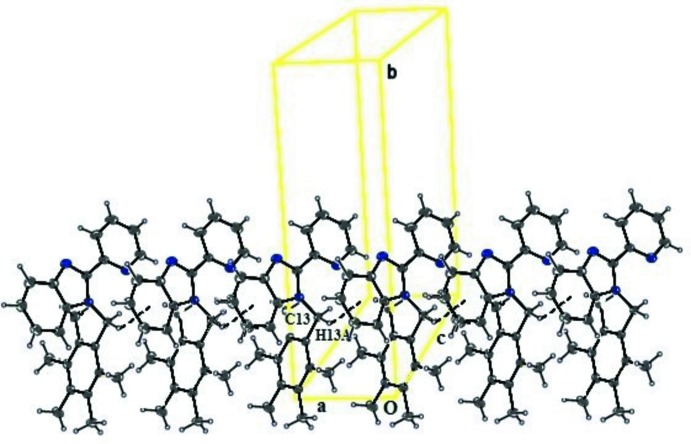
The stacking of the title compound along the a axis with C—H···π type hydrogen-bond interactions (dashed lines). Displacement ellipsoids are drawn at the 50% probability level.

## supplementary crystallographic information

### 1. Comment

The N—N type ligand system 2-(2-pyridyl)benzimidazole has a venerable history in coordination chemistry (Sahin *et al.*, 2010; Harkins *et al.*, 1956; Chiswell *et al.*, 1964; De Castro *et al.*, 1991; Maekawa *et al.*, 1994; Khalil *et al.*, 2001; Boca *et al.*, 1997). Lots of platinum chemistry bearing N—N type ligand systems involving 2,2'-bipyridine has been reported aimed at the design of drugs having less serious side-effects than those of cisplatin, or which could extend the scope of Pt based chemotherapy to tumours (Lippert, 1999; Wong & Giandomenico, 1999; Kelland & Farrell, 2000).

The molecular structure of the title compound is shown in Fig. 1. Bond lengths and angles are in good agreement with those reported for related structures (Çelik *et al.*, 2007; Çelik *et al.*, 2009; Çelik *et al.*, 2014). The benzimidazole ring system is substantially planar, the maximum deviation being 0.027 (2) Å for atom C8, and forms dihedral angles of 7.81 (5) and 87.61 (4)° with the mean planes through the pyridine (N1/C1–C5) and phenyl (C14–C19) rings, respectively. An intramolecular C—H···N hydrogen bond is present (Table 1). In the crystal structure (Fig. 2), molecules are stacked along the *a* axis by weak C—H···π hydrogen interactions (Table 1) involving the C7—C12 benzene ring of the benzimidazole moiety.

### 2. Experimental

NaH (60%) (398 mg, 11.0 mmol) was washed two times with dry hexane, filtered off *via* cannula and a solution of the 2-pyridiylbenzimidazole (1.95 g, 10.0 mmol) in dry toluene (10 ml) was added, then the solution was heated at 90°C for 24 h. Evolution of hydrogen was observed at this temperature. 2,3,4,5,6-Pentamethylbenzyl bromide (2.45 g, 10.0 mmol) was added to this mixture at room temperature and then heated again at 90°C for 1 day. Then volatiles were evaporated in vacuum to dryness. The residue was dissolved in CH_2_C_l2_ and filtered *via* cannula on celite. The desired product was obtained after concentration of CH_2_C_l2_ (15 ml) and then precipitated with hexane (30 ml). The off-white solid obtained in 86% yield. M. p. 416–418 K. ^1^H NMR (400 MHz, CDCl_3_, δ p.p.m.): 1.26 (s, 6H, *o*-(CH_3_)_2_); 1.36(s, 6H, *m*-(CH_3_)_2_); 2.13–2.20 (s, 3H, *p*-CH_3_); 5.50 (s, 2H, N—CH_2_); 6.86 (s, 1H, Ar—CH); 7.11 (s, 1H, Ar—CH); 7.26 (s, 1H, Ar—CH); 7.35 (s, 1H, Ar—CH); 7.49 (s, 1H, Ar—CH); 8.46 (s, 1H, Ar—CH); 10.19 (s, 2H, Ar—CH). ^13^C NMR (100.56 MHz, CDCl_3_, δ p.p.m.): 17.0; 17.4; 29.6; 31.7; 34.4; 35.2; 48.7; 49.3; 56.2; 117.9; 121.2; 122.4; 125.5; 126.5; 133.8; 134.0; 137.5; 140.7; 158.0; 167.9.

### 3. Refinement

All H atoms were placed geometrically and refined using a riding model approximation, with C—H = 0.93–0.97 Å, and with *U*_iso_(H) = 1.2 *U*_eq_(C) or 1.5 *U*_eq_(C) for methyl H atoms. A rotating group model was applied to the methyl groups.

### Crystal data

**Table:**

C_24_H_25_N_3_	*F*(000) = 760
*M**_r_* = 355.47	*D*_x_ = 1.242 Mg m^−^^3^
Monoclinic, *P*2_1_/*n*	Mo *K*α radiation, λ = 0.71073 Å
*a* = 5.3470 (3) Å	Cell parameters from 1276 reflections
*b* = 21.0622 (12) Å	θ = 2.3–31.5°
*c* = 17.0379 (9) Å	µ = 0.07 mm^−^^1^
β = 97.699 (3)°	*T* = 296 K
*V* = 1901.50 (18) Å^3^	Prism, yellow
*Z* = 4	0.25 × 0.20 × 0.15 mm

### Data collection

**Table:**

Bruker APEXII CCD diffractometer	3056 reflections with *I* > 2σ(*I*)
Radiation source: fine-focus sealed tube	*R*_int_ = 0.026
φ and ω scans	θ_max_ = 26.0°, θ_min_ = 2.3°
Absorption correction: multi-scan (Blessing, 1995)	*h* = −6→6
*T*_min_ = 0.982, *T*_max_ = 0.989	*k* = −25→25
25354 measured reflections	*l* = −21→20
3741 independent reflections	

### Refinement

**Table:**

Refinement on *F*^2^	Primary atom site location: structure-invariant direct methods
Least-squares matrix: full	Secondary atom site location: difference Fourier map
*R*[*F*^2^ > 2σ(*F*^2^)] = 0.052	Hydrogen site location: inferred from neighbouring sites
*wR*(*F*^2^) = 0.150	H-atom parameters constrained
*S* = 1.04	*w* = 1/[σ^2^(*F*_o_^2^) + (0.0775*P*)^2^ + 0.5927*P*] where *P* = (*F*_o_^2^ + 2*F*_c_^2^)/3
3741 reflections	(Δ/σ)_max_ < 0.001
254 parameters	Δρ_max_ = 0.24 e Å^−^^3^
0 restraints	Δρ_min_ = −0.17 e Å^−^^3^

### Special details

**Table:**

Geometry. All e.s.d.'s (except the e.s.d. in the dihedral angle between two l.s. planes) are estimated using the full covariance matrix. The cell e.s.d.'s are taken into account individually in the estimation of e.s.d.'s in distances, angles and torsion angles; correlations between e.s.d.'s in cell parameters are only used when they are defined by crystal symmetry. An approximate (isotropic) treatment of cell e.s.d.'s is used for estimating e.s.d.'s involving l.s. planes.

### Fractional atomic coordinates and isotropic or equivalent isotropic displacement parameters (Å^2^)

**Table:**

	*x*	*y*	*z*	*U*_iso_*/*U*_eq_	
C1	1.0873 (5)	0.86580 (11)	0.44231 (14)	0.0724 (6)	
H1	1.2174	0.8428	0.4246	0.087*	
C2	1.0060 (5)	0.91952 (12)	0.40152 (14)	0.0737 (6)	
H2	1.0790	0.9327	0.3577	0.088*	
C3	0.8151 (5)	0.95308 (12)	0.42706 (16)	0.0842 (7)	
H3	0.7548	0.9900	0.4011	0.101*	
C4	0.7133 (5)	0.93151 (10)	0.49165 (15)	0.0771 (7)	
H4	0.5809	0.9535	0.5094	0.092*	
C5	0.8068 (3)	0.87716 (7)	0.53054 (11)	0.0465 (4)	
C6	0.6926 (3)	0.85653 (7)	0.60059 (10)	0.0452 (4)	
C7	0.4595 (4)	0.86110 (8)	0.69313 (11)	0.0507 (4)	
C8	0.2891 (4)	0.87790 (10)	0.74483 (13)	0.0658 (6)	
H8	0.2014	0.9161	0.7387	0.079*	
C9	0.2541 (4)	0.83708 (10)	0.80460 (13)	0.0667 (6)	
H9	0.1414	0.8477	0.8396	0.080*	
C10	0.3850 (4)	0.77962 (10)	0.81392 (12)	0.0598 (5)	
H10	0.3597	0.7531	0.8558	0.072*	
C11	0.5507 (4)	0.76104 (9)	0.76284 (10)	0.0505 (4)	
H11	0.6360	0.7225	0.7690	0.061*	
C12	0.5841 (3)	0.80251 (8)	0.70185 (10)	0.0432 (4)	
C13	0.8894 (3)	0.74472 (7)	0.62583 (10)	0.0422 (4)	
H13A	1.0264	0.7410	0.6691	0.051*	
H13B	0.9632	0.7525	0.5777	0.051*	
C14	0.7459 (3)	0.68215 (7)	0.61734 (9)	0.0370 (4)	
C15	0.5434 (3)	0.67529 (7)	0.55618 (9)	0.0391 (4)	
C16	0.4047 (3)	0.61901 (8)	0.54888 (10)	0.0452 (4)	
C17	0.4753 (3)	0.56796 (8)	0.59946 (11)	0.0471 (4)	
C18	0.6838 (3)	0.57318 (7)	0.65801 (10)	0.0448 (4)	
C19	0.8190 (3)	0.63066 (7)	0.66771 (10)	0.0412 (4)	
C20	0.4783 (4)	0.72858 (9)	0.49769 (10)	0.0511 (4)	
H20A	0.406 (3)	0.7111 (2)	0.4472 (7)	0.077*	
H20B	0.630 (2)	0.7521 (5)	0.4912 (6)	0.077*	
H20C	0.357 (2)	0.7567 (5)	0.5172 (5)	0.077*	
C21	0.1762 (4)	0.61342 (11)	0.48609 (14)	0.0703 (6)	
H21A	0.2254 (10)	0.5965 (8)	0.4399 (8)	0.105*	
H21B	0.106 (2)	0.6535 (6)	0.4754 (8)	0.105*	
H21C	0.057 (2)	0.5868 (8)	0.5043 (5)	0.105*	
C22	0.3235 (5)	0.50692 (10)	0.59025 (15)	0.0710 (6)	
H22A	0.314 (3)	0.4923 (5)	0.5373 (9)	0.107*	
H22B	0.158 (3)	0.5146 (2)	0.6025 (10)	0.107*	
H22C	0.403 (2)	0.4757 (5)	0.6251 (9)	0.107*	
C23	0.7616 (5)	0.51661 (9)	0.71064 (13)	0.0655 (6)	
H23A	0.626 (2)	0.5047 (5)	0.7383 (8)	0.098*	
H23B	0.904 (3)	0.5276 (3)	0.7476 (8)	0.098*	
H23C	0.803 (3)	0.4820 (6)	0.6790 (5)	0.098*	
C24	1.0435 (4)	0.63574 (10)	0.73176 (13)	0.0630 (5)	
H24A	1.168 (2)	0.6592 (7)	0.7134 (4)	0.094*	
H24B	1.104 (2)	0.5960 (6)	0.7452 (7)	0.094*	
H24C	0.9950 (10)	0.6551 (7)	0.7754 (7)	0.094*	
N1	0.9926 (3)	0.84413 (8)	0.50574 (10)	0.0616 (4)	
N2	0.5315 (3)	0.89400 (7)	0.63031 (10)	0.0561 (4)	
N3	0.7309 (3)	0.79969 (6)	0.64092 (8)	0.0406 (3)	

### Atomic displacement parameters (Å^2^)

**Table:**

	*U*^11^	*U*^22^	*U*^33^	*U*^12^	*U*^13^	*U*^23^
C1	0.0797 (15)	0.0651 (13)	0.0780 (14)	0.0052 (11)	0.0312 (12)	0.0205 (11)
C2	0.0844 (16)	0.0737 (14)	0.0641 (13)	−0.0104 (12)	0.0138 (12)	0.0232 (11)
C3	0.1041 (19)	0.0620 (14)	0.0871 (17)	0.0111 (13)	0.0152 (15)	0.0351 (13)
C4	0.0940 (17)	0.0516 (12)	0.0889 (16)	0.0184 (11)	0.0244 (14)	0.0216 (11)
C5	0.0539 (10)	0.0329 (8)	0.0517 (10)	−0.0066 (7)	0.0031 (8)	0.0006 (7)
C6	0.0522 (10)	0.0305 (8)	0.0519 (10)	−0.0019 (7)	0.0034 (8)	−0.0032 (7)
C7	0.0580 (11)	0.0396 (9)	0.0551 (10)	−0.0025 (8)	0.0099 (9)	−0.0119 (8)
C8	0.0756 (14)	0.0522 (11)	0.0726 (13)	0.0050 (10)	0.0212 (11)	−0.0188 (10)
C9	0.0737 (14)	0.0657 (13)	0.0651 (13)	−0.0053 (11)	0.0261 (11)	−0.0210 (11)
C10	0.0732 (13)	0.0590 (11)	0.0490 (10)	−0.0122 (10)	0.0152 (9)	−0.0084 (9)
C11	0.0582 (11)	0.0457 (9)	0.0476 (10)	−0.0050 (8)	0.0070 (8)	−0.0042 (7)
C12	0.0451 (9)	0.0397 (8)	0.0442 (9)	−0.0072 (7)	0.0038 (7)	−0.0087 (7)
C13	0.0405 (9)	0.0345 (8)	0.0514 (9)	0.0012 (6)	0.0059 (7)	0.0014 (7)
C14	0.0395 (8)	0.0319 (7)	0.0407 (8)	0.0008 (6)	0.0096 (6)	−0.0031 (6)
C15	0.0416 (8)	0.0382 (8)	0.0389 (8)	0.0030 (6)	0.0108 (7)	−0.0045 (6)
C16	0.0441 (9)	0.0449 (9)	0.0479 (9)	−0.0036 (7)	0.0108 (7)	−0.0122 (7)
C17	0.0536 (10)	0.0381 (9)	0.0538 (10)	−0.0077 (7)	0.0227 (8)	−0.0116 (7)
C18	0.0574 (10)	0.0339 (8)	0.0473 (9)	0.0041 (7)	0.0230 (8)	−0.0003 (7)
C19	0.0453 (9)	0.0367 (8)	0.0426 (8)	0.0044 (7)	0.0094 (7)	0.0000 (6)
C20	0.0573 (11)	0.0504 (10)	0.0441 (9)	0.0048 (8)	0.0010 (8)	0.0003 (8)
C21	0.0589 (12)	0.0747 (14)	0.0738 (14)	−0.0118 (11)	−0.0033 (11)	−0.0163 (11)
C22	0.0808 (15)	0.0505 (11)	0.0862 (15)	−0.0231 (10)	0.0274 (12)	−0.0111 (10)
C23	0.0940 (16)	0.0398 (10)	0.0665 (13)	0.0052 (10)	0.0247 (11)	0.0093 (9)
C24	0.0675 (13)	0.0540 (11)	0.0624 (12)	0.0037 (9)	−0.0094 (10)	0.0093 (9)
N1	0.0691 (10)	0.0521 (9)	0.0671 (10)	0.0057 (8)	0.0216 (8)	0.0180 (8)
N2	0.0697 (10)	0.0346 (7)	0.0650 (10)	0.0040 (7)	0.0129 (8)	−0.0039 (7)
N3	0.0456 (7)	0.0305 (6)	0.0455 (7)	−0.0029 (5)	0.0056 (6)	−0.0021 (5)

### Geometric parameters (Å, º)

**Table:**

C1—N1	1.334 (3)	C13—H13B	0.9700
C1—C2	1.368 (3)	C14—C19	1.405 (2)
C1—H1	0.9300	C14—C15	1.406 (2)
C2—C3	1.360 (4)	C15—C16	1.395 (2)
C2—H2	0.9300	C15—C20	1.510 (2)
C3—C4	1.369 (3)	C16—C17	1.398 (3)
C3—H3	0.9300	C16—C21	1.517 (3)
C4—C5	1.382 (3)	C17—C18	1.397 (3)
C4—H4	0.9300	C17—C22	1.517 (2)
C5—N1	1.328 (2)	C18—C19	1.408 (2)
C5—C6	1.477 (3)	C18—C23	1.515 (2)
C6—N2	1.319 (2)	C19—C24	1.514 (3)
C6—N3	1.382 (2)	C20—H20A	0.968 (12)
C7—N2	1.373 (2)	C20—H20B	0.968 (13)
C7—C8	1.395 (3)	C20—H20C	0.968 (12)
C7—C12	1.401 (2)	C21—H21A	0.933 (14)
C8—C9	1.365 (3)	C21—H21B	0.933 (14)
C8—H8	0.9300	C21—H21C	0.933 (14)
C9—C10	1.396 (3)	C22—H22A	0.948 (14)
C9—H9	0.9300	C22—H22B	0.948 (14)
C10—C11	1.379 (3)	C22—H22C	0.948 (14)
C10—H10	0.9300	C23—H23A	0.951 (14)
C11—C12	1.387 (2)	C23—H23B	0.951 (14)
C11—H11	0.9300	C23—H23C	0.951 (13)
C12—N3	1.384 (2)	C24—H24A	0.915 (14)
C13—N3	1.478 (2)	C24—H24B	0.915 (13)
C13—C14	1.522 (2)	C24—H24C	0.915 (13)
C13—H13A	0.9700		
			
N1—C1—C2	124.4 (2)	C15—C16—C17	120.14 (16)
N1—C1—H1	117.8	C15—C16—C21	119.82 (17)
C2—C1—H1	117.8	C17—C16—C21	120.05 (16)
C3—C2—C1	117.9 (2)	C18—C17—C16	120.23 (15)
C3—C2—H2	121.0	C18—C17—C22	120.34 (17)
C1—C2—H2	121.0	C16—C17—C22	119.42 (18)
C2—C3—C4	118.7 (2)	C17—C18—C19	119.95 (15)
C2—C3—H3	120.6	C17—C18—C23	119.35 (16)
C4—C3—H3	120.6	C19—C18—C23	120.69 (17)
C3—C4—C5	120.2 (2)	C14—C19—C18	119.69 (15)
C3—C4—H4	119.9	C14—C19—C24	120.95 (15)
C5—C4—H4	119.9	C18—C19—C24	119.35 (15)
N1—C5—C4	121.30 (19)	C15—C20—H20A	109.5
N1—C5—C6	120.74 (15)	C15—C20—H20B	109.5
C4—C5—C6	117.96 (18)	H20A—C20—H20B	109.5
N2—C6—N3	112.86 (16)	C15—C20—H20C	109.5
N2—C6—C5	119.77 (15)	H20A—C20—H20C	109.5
N3—C6—C5	127.37 (15)	H20B—C20—H20C	109.5
N2—C7—C8	129.74 (18)	C16—C21—H21A	109.5
N2—C7—C12	110.39 (16)	C16—C21—H21B	109.5
C8—C7—C12	119.87 (18)	H21A—C21—H21B	109.5
C9—C8—C7	118.6 (2)	C16—C21—H21C	109.5
C9—C8—H8	120.7	H21A—C21—H21C	109.5
C7—C8—H8	120.7	H21B—C21—H21C	109.5
C8—C9—C10	120.93 (19)	C17—C22—H22A	109.5
C8—C9—H9	119.5	C17—C22—H22B	109.5
C10—C9—H9	119.5	H22A—C22—H22B	109.5
C11—C10—C9	121.92 (19)	C17—C22—H22C	109.5
C11—C10—H10	119.0	H22A—C22—H22C	109.5
C9—C10—H10	119.0	H22B—C22—H22C	109.5
C10—C11—C12	116.88 (18)	C18—C23—H23A	109.5
C10—C11—H11	121.6	C18—C23—H23B	109.5
C12—C11—H11	121.6	H23A—C23—H23B	109.5
N3—C12—C11	132.66 (16)	C18—C23—H23C	109.5
N3—C12—C7	105.53 (15)	H23A—C23—H23C	109.5
C11—C12—C7	121.80 (17)	H23B—C23—H23C	109.5
N3—C13—C14	113.67 (13)	C19—C24—H24A	109.5
N3—C13—H13A	108.8	C19—C24—H24B	109.5
C14—C13—H13A	108.8	H24A—C24—H24B	109.5
N3—C13—H13B	108.8	C19—C24—H24C	109.5
C14—C13—H13B	108.8	H24A—C24—H24C	109.5
H13A—C13—H13B	107.7	H24B—C24—H24C	109.5
C19—C14—C15	119.78 (14)	C5—N1—C1	117.39 (17)
C19—C14—C13	120.99 (14)	C6—N2—C7	105.26 (15)
C15—C14—C13	119.19 (14)	C6—N3—C12	105.94 (13)
C16—C15—C14	120.06 (15)	C6—N3—C13	129.98 (14)
C16—C15—C20	119.99 (15)	C12—N3—C13	124.08 (13)
C14—C15—C20	119.95 (14)		
			
N1—C1—C2—C3	−0.2 (4)	C15—C16—C17—C22	−179.52 (16)
C1—C2—C3—C4	−0.3 (4)	C21—C16—C17—C22	0.7 (3)
C2—C3—C4—C5	1.0 (4)	C16—C17—C18—C19	1.9 (2)
C3—C4—C5—N1	−1.2 (4)	C22—C17—C18—C19	−178.06 (16)
C3—C4—C5—C6	179.1 (2)	C16—C17—C18—C23	−178.23 (16)
N1—C5—C6—N2	171.36 (17)	C22—C17—C18—C23	1.8 (2)
C4—C5—C6—N2	−9.0 (3)	C15—C14—C19—C18	−1.9 (2)
N1—C5—C6—N3	−9.7 (3)	C13—C14—C19—C18	−179.49 (14)
C4—C5—C6—N3	169.96 (19)	C15—C14—C19—C24	177.28 (16)
N2—C7—C8—C9	−178.4 (2)	C13—C14—C19—C24	−0.3 (2)
C12—C7—C8—C9	1.9 (3)	C17—C18—C19—C14	−1.2 (2)
C7—C8—C9—C10	−0.1 (3)	C23—C18—C19—C14	178.93 (15)
C8—C9—C10—C11	−1.2 (3)	C17—C18—C19—C24	179.62 (16)
C9—C10—C11—C12	0.7 (3)	C23—C18—C19—C24	−0.3 (2)
C10—C11—C12—N3	−179.82 (17)	C4—C5—N1—C1	0.7 (3)
C10—C11—C12—C7	1.1 (3)	C6—C5—N1—C1	−179.67 (18)
N2—C7—C12—N3	−1.48 (19)	C2—C1—N1—C5	0.1 (4)
C8—C7—C12—N3	178.23 (17)	N3—C6—N2—C7	0.2 (2)
N2—C7—C12—C11	177.79 (16)	C5—C6—N2—C7	179.26 (15)
C8—C7—C12—C11	−2.5 (3)	C8—C7—N2—C6	−178.8 (2)
N3—C13—C14—C19	−121.53 (16)	C12—C7—N2—C6	0.8 (2)
N3—C13—C14—C15	60.88 (19)	N2—C6—N3—C12	−1.10 (19)
C19—C14—C15—C16	4.3 (2)	C5—C6—N3—C12	179.91 (16)
C13—C14—C15—C16	−178.05 (14)	N2—C6—N3—C13	178.34 (15)
C19—C14—C15—C20	−174.84 (15)	C5—C6—N3—C13	−0.7 (3)
C13—C14—C15—C20	2.8 (2)	C11—C12—N3—C6	−177.65 (18)
C14—C15—C16—C17	−3.6 (2)	C7—C12—N3—C6	1.50 (17)
C20—C15—C16—C17	175.52 (15)	C11—C12—N3—C13	2.9 (3)
C14—C15—C16—C21	176.10 (16)	C7—C12—N3—C13	−177.98 (14)
C20—C15—C16—C21	−4.7 (2)	C14—C13—N3—C6	−125.33 (17)
C15—C16—C17—C18	0.5 (2)	C14—C13—N3—C12	54.0 (2)
C21—C16—C17—C18	−179.21 (16)		

### Hydrogen-bond geometry (Å, º)

Cg is the centroid of the C7–C12 benzene ring.

**Table:**

*D*—H···*A*	*D*—H	H···*A*	*D*···*A*	*D*—H···*A*
C13—H13*A*···*Cg*^i^	0.97	2.91	3.6941 (18)	139
C13—H13*B*···N1	0.97	2.30	3.029 (2)	131

Symmetry code: (i) *x*−1, *y*, *z*.
